# The effects of dietary essential fatty acid ratios and linoleic acid level in grow–finish pigs

**DOI:** 10.1093/jas/skad263

**Published:** 2023-08-04

**Authors:** Spenser L Becker, Dalton C Humphrey, Locke A Karriker, Justin T Brown, Kristin J Skoland, Laura L Greiner

**Affiliations:** Department of Animal Science, Iowa State University, Ames, IA 50011, USA; Department of Animal Science, Iowa State University, Ames, IA 50011, USA; Swine Medicine Education Center, College of Veterinary Medicine, Iowa State University, Ames, IA 50011, USA; Swine Medicine Education Center, College of Veterinary Medicine, Iowa State University, Ames, IA 50011, USA; Swine Medicine Education Center, College of Veterinary Medicine, Iowa State University, Ames, IA 50011, USA; Department of Animal Science, Iowa State University, Ames, IA 50011, USA

**Keywords:** essential fatty acids, grow–finish pigs, inflammation, joint, linoleic acid, synovial fluid

## Abstract

The objective of this study was to investigate the effects of dietary linoleic acid level and the ratio of linoleic acid:linolenic acid (**LA:ALA**) on the growth performance, expression of genes associated with lipid metabolism, and inflammatory status of grow–finish pigs. A total of 300 growing pigs (body weight [**BW**] = 41.1 ± 6.3 kg) were randomly assigned to either a high (30 g/kg; HLA) or low (15 g/kg; LLA) dietary linoleic acid level with a high (23:1; HR), moderate (13:1; MR) or low (4:1; LR) dietary LA:ALA in a 2 × 3 factorial design. Diets were fed across three 28-d phases and were balanced for dietary metabolizable energy. Pigs were housed five pigs per pen in single-sex pens. Blood samples were collected on days 0, 21, 42, and 84, and synovial fluid was collected from the hock joint on days 0 and 84 for inflammatory marker analysis. Data were analyzed as repeated measures using PROC MIXED (SAS 9.4) with initial BW as a covariate, pen as the experimental unit, and LA level, LA:ALA, sex, phases, and their interactions as fixed effects. Compared to HLA, LLA pigs tended to have increased BW at days 56 and 84 (*P *= 0.088). There was no effect of LA × LA:ALA for growth performance. For the overall days 0 to 84 growth period, pigs fed HR had increased ADG compared to MR, with pigs receiving LR performing intermediate of MR and HR. Gilts receiving HR diets had increased day 84 BW compared to gilts receiving the low and moderate LA:ALA (*P *= 0.006), which was a result of improved overall days 0 to 84 ADG compared to gilts receiving the MR diets (*P =* 0.023). Barrows fed LR had improved BW on day 56 compared to MR and HR and higher final BW compared to HR, with MR performing intermediately (*P *= 0.006). This was a result of greater days 0 to 84 ADG (*P *= 0.023). Overall, C-reactive protein (**CRP**), tumor necrosis factor-α (**TNFα**), and interleukin-6 were reduced in the plasma of pigs over time (*P ≤ *0.037). Across all treatments, CRP and TNFα were reduced in the hock and carpus synovial fluid on day 84 vs. day 0 (*P ≤ *0.049). In conclusion, LA:ALA ratios utilized in this study can be fed at varying linoleic acid levels without impacting growth or inflammation. Additionally, LA:ALA ratios can differentially impact the growth of gilts and barrows.

## Teaser

Differing dietary essential fatty acid ratios can alter the growth performance of barrows and gilts during the grow–finish phase.

## Introduction

Dietary polyunsaturated fatty acid (**PUFA**) supplementation has been extensively studied across species. The most common, naturally occurring PUFA groups are 18 to 20 carbons, which can be classified into three main families: n-3, n-6, and n-9. Many mammals can synthesize saturated fatty acids (**SFA**), along with a few monounsaturated fatty acids (**MUFA**) from carbohydrates and proteins. They are unable to synthesize n-6 PUFA and n-3 PUFA from 18-carbon MUFA (oleic acid; 18:1n-9) because they lack the desaturase enzymes required to introduce a *cis* double bond at the n-3 or n-6 positions. Therefore, linoleic acid (**LA**: C18:2n-6) and α-linolenic acid (**ALA**: C18:3n-3) are essential fatty acids (**EFA**) and need to be provided in the diet. The requirement for fatty acids (**FAs**) in animal diets was first described in rat studies, where a fat-free diet resulted in various signs of deficiency (Burr and Burr, 1929). Though deficiency across species is rarely observed, low dietary LA and ALA intakes contribute to several disorders and diseases (Holman, 1992; Campbell, 1993; Reinwald et al., 2004). Many plant, algae, and fungi species have the necessary enzymes for converting LA to ALA, and thus serve as excellent dietary sources of LA and ALA (Harwood, 1996). Vegetable oils, including corn and soybean oils, are major sources of LA, while high quantities of ALA are found in flaxseed oil (NRC, 2012). Animal fats, including tallow and choice white grease, have a minimal content of LA and ALA and mainly consist of MUFA and SFA.

In the swine industry, lameness is a large contributor to early culling in the finishing phase, gilt development, and the sow farm. Lameness can be attributed to a variety of issues, including pathogen infections, osteoarthritis, leg conformation defects, and injury or trauma. Literature in other species has demonstrated the ability of n-3 PUFA to improve osteoarthritis (Knott et al., 2011; Moreau et al., 2012) and moderate local catabolic factors of bone (Watkins, 2003; Shen et al., 2006; Robison and Orth, 2015). Additionally, a high n-6:n-3 has been positively associated with a reduced rate of bone formation (Watkins et al., 2000), while a lower n-6:n-3 has been shown to modulate bone prostaglandin production (Al-Nouri et al., 2012) and reduce fracture incidence (Toscano et al., 2015). A dietary n-6:n-3 ranging from 1:1 to 5:1 has also been shown to modulate lipid metabolism and inflammation in pigs (Duan et al., 2014; Szostak et al., 2016).

Many studies in swine and other species utilize marine oil sources when evaluating impacts of n-3 supplementation or to lower the ratio of n-6:n-3; however, it should be noted that these oils represent sources of eicosapentaenoic acid (**EPA**; 20:5n-3) and docosahexaenoic acid (**DHA**; 22:6n-3) and contain low levels of ALA. Furthermore, literature specifically evaluating LA in swine diets often discusses conjugated LA (**CLA**) rather than sources such as corn or soybean oils. A study by Rosero et al. (2016) suggests a minimum of 100 g/d of LA and 10 g/d of ALA for lactating sows to avoid a negative EFA balance during lactation. In this study, EFA supplementation was provided using plant-based lipid sources and a positive effect for culling rate was seen in first parity sows (Rosero et al., 2016). Minimal data exists in growing pigs regarding dietary LA inclusion and LA:ALA ratios, and there is currently no dietary recommendation for growing pigs (NRC, 2012). Furthermore, data from human and canine literature suggests that the optimal dietary LA:ALA is approximately 4:1 (Calder, 2008). Previous work by Becker et al. (2023) evaluated dietary LA:ALA in growing pigs and found improvements in gilt growth performance when fed a 13:1 LA:ALA; however, this study did not evaluate LA:ALA ratios below 13:1. Therefore, the objective of this study was to investigate the effects of three dietary LA:ALA ratios and linoleic acid level on the growth performance, expression of genes associated with lipid metabolism and inflammation in grow–finish pigs.

## Materials and Methods

All experimental procedures adhered to guidelines for the ethical and humane use of animals for research according to the Guide for the Care and Use of Agricultural Animals in Research and Teaching (FASS, 2010) and were approved by the Iowa State University Institutional Animal Care and Use Committee (IACUC #19-354).

### Animals and experimental design

A total of 300 growing pigs (41.1 ± 6.3 kg body weight [**BW**; PIC Genus 337 × 1050, Hendersonville, TN]) were individually weighed and allotted to single-sex pens with either five barrows or five gilts per pen, for a total of 60 pens. Pens had partially slated, concrete flooring with dimensions of 1.83 m × 1.93 m. A two-space galvanized steel feeder (width = 76 cm) with hinged lids and two nipple drinkers was used to provide ad libitum access to feed and water throughout the study. Pigs were housed at the Iowa State University Swine Nutrition Research farm. Pigs originated from a porcine reproductive and respiratory syndrome virus-vaccinated herd. One pig per pen was vaccinated for *Lawsonia intercellularis* (Porcilis ILEITIS; Merck Animal Health, Madison, New Jersey), porcine circovirus type 2 and *Mycoplasma hyopneumoniae* (Circumvent PCV-M G2; Merck Animal Health) according to the manufacturer’s recommendations. Vaccines were administered to replicate commercial production practices during the gilt development period. Pig health was monitored daily. All pigs were observed and evaluated daily in the pen for signs of lameness, including the inability to bear weight on all four legs, abnormal gait or standing posture, and physical evidence of trauma, such as swelling and malformation of the leg or joint.

### Dietary treatments

Pens were randomly assigned to diets containing either a high (30 g/kg; **HLA**) or low (15 g/kg; **LLA**) linoleic acid level in combination with a high (20:1; **HR**), moderate (12:1; **MR**) or low (4:1; **LR**) LA:ALA in a 3 × 2 factorial design. Diets were fed across three 28-d phases and were balanced for dietary metabolizable energy (**ME**). Diets were presented in mash form and primarily based on corn and soybean meal (Tables 1 to 3). The diets were formulated to meet or exceed NRC (2012) nutrient recommendations of growing pigs and did not contain antibiotics or pharmaceutical levels of copper or zinc. Feed samples were collected at the completion of mixing for each phase of diet. Samples were then stored at −20 °C for subsequent analysis.

**Table 1.** Ingredient and nutrient composition of the grow–finish experimental diets to evaluate dietary essential fatty acid ratios and linoleic acid level, phase 1 (as-fed basis)

Item	30 g/kg linoleic acid			15 g/kg linoleic acid		
	20:1^1^	12:1	4:1	20:1	12:1	4:1
Ingredient, %						
Corn	70.77	70.79	70.88	70.55	70.14	70.19
Soybean meal	20.93	20.93	20.93	20.93	20.93	20.93
Beef tallow	0.00	0.00	0.00	2.85	3.35	3.36
Choice white grease	1.25	1.04	0.00	1.75	1.57	1.04
Corn oil	3.04	3.04	3.04	0.00	0.00	0.00
Flaxseed oil	0.11	0.30	1.24	0.00	0.09	0.56
DL-methionine	0.15	0.15	0.15	0.15	0.16	0.16
L-lysine HCL	0.50	0.50	0.50	0.49	0.50	0.50
L-threonine	0.19	0.19	0.19	0.18	0.19	0.19
L-tryptophan	0.03	0.03	0.03	0.03	0.03	0.03
L-valine	0.08	0.08	0.08	0.10	0.08	0.08
Premix^2^	0.35	0.35	0.35	0.35	0.35	0.35
Limestone	0.97	0.97	0.96	0.96	0.96	0.96
Monocalcium phosphate	1.14	1.14	1.14	1.14	1.14	1.14
Salt	0.50	0.50	0.50	0.50	0.50	0.50
Calculated nutrient levels^3^						
ME, Mcal/kg	3.47	3.47	3.47	3.47	3.47	3.47
Ether extract, %	3.20	3.20	3.21	3.20	3.18	3.18
Linoleic acid, %	3.00	3.00	3.00	1.50	1.50	1.50
α-Linolenic acid, %	0.75	0.25	0.15	0.38	0.13	0.08
Total P, %	0.58	0.58	0.58	0.58	0.58	0.58
STTD P, %	0.35	0.35	0.35	0.35	0.35	0.35
Calcium, %	0.70	0.70	0.70	0.70	0.70	0.70
SID Lysine, %	1.08	1.08	1.08	1.07	1.08	1.08
SID Met + Cys, %	0.61	0.61	0.61	0.61	0.61	0.61
SID Thr, %	0.67	0.67	0.67	0.67	0.67	0.67
SID Trp, %	0.19	0.19	0.19	0.19	0.19	0.19
SID Lys/ME (g/kg)/Mcal	3.10	3.10	3.10	3.10	3.10	3.10
Analyzed nutrient levels^4^						
Dry matter, %	87.18	87.36	87.48	87.18	87.52	87.40
GE, Mcal/kg	4.44	4.44	4.47	4.49	4.46	4.47
Crude protein, %	15.58	14.67	15.33	15.11	15.83	15.54
aEE, %	7.95	8.06	8.25	8.36	8.29	8.10

^1^Dietary fatty acid ratio as dietary linoleic acid:linolenic acid (LA:ALA): 20:1, 12:1, and 4:1.

^2^Provided 6,614 IU vitamin A, 827 IU vitamin D, 26 IU vitamin E, 2.6 mg vitamin K, 29.8 mg niacin, 16.5 mg pantothenic acid, 5.0 mg riboflavin, and 0.023 mg vitamin B12, 165 mg Zn (zinc sulfate), 165 mg Fe (iron sulfate), 39 mg Mn (manganese sulfate), 17 mg Cu (copper sulfate), 0.3 mg I (calcium iodate), and 0.3 mg Se (sodium selenite) per kilogram of diet.

^3^ME, metabolizable energy; STTD, standardized total tract digestible; SID, standardized ileal digestible.

^4^GE, gross energy; aEE, acid ether extract.

**Table 2.** Ingredient and nutrient composition of the grow–finish experimental diets to evaluate dietary essential fatty acid ratios and linoleic acid level, phase 2 (as-fed basis)

Item	30 g/kg linoleic acid			15 g/kg linoleic acid		
	20:1^1^	12:1	4:1	20:1	12:1	4:1
Ingredient, %						
Corn	76.41	76.32	76.30	75.70	75.65	75.92
Soybean meal	15.51	15.51	15.51	15.51	15.51	15.51
Beef tallow	0.00	0.00	0.00	3.87	3.86	3.53
Choice white grease	0.00	1.04	1.24	0.46	0.98	1.14
Corn oil	2.94	2.94	2.94	0.00	0.00	0.00
Flaxseed oil	0.12	0.30	1.25	0.00	0.09	0.57
dl-Methionine	0.11	0.11	0.11	0.11	0.11	0.11
l-lysine HCL	0.47	0.47	0.47	0.47	0.47	0.47
l-Threonine	0.17	0.17	0.17	0.17	0.17	0.17
l-Tryptophan	0.04	0.04	0.04	0.04	0.04	0.04
l-Valine	0.06	0.06	0.06	0.06	0.06	0.08
Premix^2^	0.35	0.35	0.35	0.35	0.35	0.35
Limestone	0.98	0.98	0.98	0.98	0.98	0.98
Monocalcium phosphate	1.21	1.21	1.21	1.21	1.21	1.21
Salt	0.50	0.50	0.50	0.50	0.50	0.50
Calculated nutrient levels^3^						
ME, Mcal/kg	3.47	3.47	3.47	3.47	3.47	3.46
Ether extract, %	3.26	3.25	3.25	3.23	3.23	3.24
Linoleic acid, %	3.00	3.00	3.00	1.50	1.50	1.50
α-Linolenic acid, %	0.75	0.25	0.15	0.38	0.13	0.08
Total P, %	0.57	0.57	0.57	0.57	0.57	0.57
STTD P, %	0.35	0.35	0.35	0.35	0.35	0.35
Calcium, %	0.70	0.70	0.70	0.70	0.70	0.70
SID Lysine, %	0.92	0.92	0.92	0.92	0.92	0.92
SID Met + Cys, %	0.52	0.52	0.52	0.52	0.52	0.52
SID Thr, %	0.58	0.58	0.58	0.58	0.58	0.58
SID Trp, %	0.17	0.17	0.17	0.17	0.17	0.17
SID Lys/ME (g/kg)/Mcal	2.65	2.65	2.65	2.65	2.65	2.65
Analyzed nutrient levels^4^						
Dry matter, %	87.45	87.40	87.42	87.55	87.22	87.25
GE, Mcal/kg	4.42	4.42	4.43	4.49	4.47	4.49
Crude protein, %	13.14	12.74	12.82	13.23	13.26	13.33
aEE, %	7.95	8.06	8.25	8.36	8.29	8.10

^1^Dietary fatty acid ratio as dietary linoleic acid:linolenic acid (LA:ALA): 20:1, 12:1, and 4:1.

^2^Provided 6,125 IU vitamin A, 700 IU vitamin D, 50 IU vitamin E, 3.0 mg vitamin K, 56.0 mg niacin, 27.0 mg pantothenic acid, 11.0 mg riboflavin, and 0.050 mg vitamin B12, 165 mg Zn (zinc sulfate), 165 mg Fe (iron sulfate), 39 mg Mn (manganese sulfate), 16.5 mg Cu (copper sulfate), 0.3 mg I (calcium iodate), and 0.3 mg Se (sodium selenite) per kg of diet.

^3^ME, metabolizable energy; STTD, standardized total tract digestible; SID, standardized ileal digestible.

^4^GE, gross energy; aEE, acid ether extract.

**Table 3.** Ingredient and nutrient composition of the grow–finish experimental diets to evaluate dietary essential fatty acid ratios and linoleic acid level, phase 3 (as-fed basis)

Item	30 g/kg linoleic acid			15 g/kg linoleic acid		
	20:1^1^	12:1	4:1	20:1	12:1	4:1
Ingredient, %						
Corn	79.21	79.23	79.32	78.72	78.56	78.60
Soybean meal	12.82	12.82	12.82	12.82	12.82	12.82
Beef tallow	0.00	0.00	0.00	3.91	4.12	4.13
Choice white grease	1.23	1.02	0.00	0.80	0.67	0.14
Corn oil	2.89	2.89	2.88	0.00	0.00	0.00
Flaxseed oil	0.12	0.31	1.25	0.00	0.09	0.57
dl-Methionine	0.07	0.07	0.07	0.07	0.07	0.07
l-Lysine HCL	0.40	0.40	0.40	0.40	0.40	0.40
l-Threonine	0.13	0.13	0.13	0.13	0.13	0.13
l-Tryptophan	0.03	0.03	0.03	0.03	0.03	0.03
l-Valine	0.03	0.03	0.02	0.04	0.03	0.03
Premix^2^	0.35	0.35	0.35	0.35	0.35	0.35
Limestone	0.98	0.98	0.98	0.98	0.98	0.98
Monocalcium phosphate	1.24	1.24	1.24	1.25	1.25	1.25
Salt	0.50	0.50	0.50	0.50	0.50	0.50
Calculated nutrient levels^3^						
ME, Mcal/kg	3.48	3.48	3.48	3.48	3.48	3.47
Ether extract, %	3.29	3.28	3.28	3.26	3.26	3.26
Linoleic acid, %	3.00	3.00	3.00	1.50	1.50	1.50
α-Linolenic acid, %	0.75	0.25	0.15	0.38	0.13	0.08
Total P, %	0.56	0.56	0.56	0.56	0.56	0.56
STTD P, %	0.35	0.35	0.35	0.35	0.35	0.35
Calcium, %	0.70	0.70	0.70	0.70	0.70	0.70
SID Lysine, %	0.80	0.80	0.80	0.80	0.80	0.80
SID Met + Cys, %	0.46	0.46	0.46	0.46	0.46	0.45
SID Thr, %	0.50	0.50	0.50	0.50	0.50	0.50
SID Trp, %	0.14	0.14	0.14	0.14	0.14	0.14
SID Lys/ME (g/kg)/Mcal	2.30	2.30	2.30	2.30	2.30	2.30
Analyzed nutrient levels^4^						
Dry matter, %	87.68	87.37	87.15	87.10	87.24	87.65
GE, Mcal/kg	4.42	4.42	4.41	4.43	4.43	4.41
Crude protein, %	11.52	12.29	12.19	11.78	12.10	12.53
aEE, %	7.95	8.06	8.25	8.36	8.29	8.10

^1^Dietary fatty acid ratio as dietary linoleic acid:linolenic acid (LA:ALA): 20:1, 12:1, and 4:1.

^2^Provided 6,125 IU vitamin A, 700 IU vitamin D, 50 IU vitamin E, 3.0 mg vitamin K, 56.0 mg niacin, 27.0 mg pantothenic acid, 11.0 mg riboflavin, and 0.05 mg vitamin B12, 165 mg Zn (zinc sulfate), 165 mg Fe (iron sulfate), 39 mg Mn (manganese sulfate), 17 mg Cu (copper sulfate), 0.3 mg I (calcium iodate), and 0.3 mg Se (sodium selenite) per kilogram of diet.

^3^ME, metabolizable energy; STTD, standardized total tract digestible; SID, standardized ileal digestible.

^4^GE, gross energy; aEE, acid ether extract.

### Sample collection

Pigs and feeders were individually weighed on days 0, 28, 56, and 84. Feed disappearance was recorded to calculate average daily gain (**ADG**), average daily feed intake (**ADFI**), and gain-to-feed ratio (**G:F**) for each phase. On days 0, 21, 42, and 84, blood samples were collected from three vaccinated barrows and three vaccinated gilts per treatment via sterile, jugular venipuncture into an ethylenediaminetetraacetic acid (EDTA) treated vacutainer tube (Becton Dickinson, Franklin Lakes, NJ). Plasma was separated by centrifugation (2,000 × *g* for 10 min at 4 °C), divided into two aliquots, and stored at −80 °C for further analysis.

Synovial fluid was collected according to methods described by (Canning et al., 2018). Briefly, the same subset of 36 pigs received a single intramuscular injection of tiletamine hydrochloride and zolazepam hydrochloride (4.4 mg/kg), ketamine hydrochloride (2.2 mg/kg), and xylazine hydrochloride (4.4 mg/kg). Each pig was fully anesthetized and had a negative palpebral response and toe withdrawal response prior to collection of synovial fluid. After sedation was ­confirmed, each pig was monitored closely during the collection procedure by recording respiration and heart rates and rectal temperature at least once every 10 min during sedation and at 30-min to 1-h intervals during recovery. Animals were monitored until they were able to stand unassisted.

Once anesthetized, each pig was positioned in dorsal recumbency. One tarsus and carpus of each pig were randomly selected, shaved, and further prepared with a 2% chlorhexidine soap scrub (VetOne, Boise, ID), a 70% alcohol scrub, and final application of a tincture of 2% chlorhexidine. The chlorhexidine soap and alcohol scrubs were repeated once to ensure asepsis of the centesis sites. To aspirate the synovial fluid, a sterile 18-gauge, 1.5-inch needle attached to a 12-mL sterile syringe was inserted into the dorsolateral aspect of the carpus or tarsus, and negative pressure was used to aspirate synovial fluid into the syringe. Given the continuity between joint spaces within the tarsal and carpal joints, needle insertion at the same location in each joint was attempted. Heart rate, respiratory rate, rectal temperature, and depth of sedation were monitored at least once every 10 min while the pig was anesthetized and at 30-min to 1-h intervals during recovery from anesthesia.

On day 84, the same subset of three barrows and three gilts per treatment were euthanized by captive bolt stunning followed by exsanguination. A 5-cm^2^ section of adipose tissue from the tenth rib and a 5-cm^2^ section of liver from the right lateral lobe were removed, snap frozen in liquid N and stored at −80 °C for later analysis. One carpus and one hock joint were opened for collection of synovial fluid. Synovial fluid samples were stored at −80 °C for later analysis.

### Chemical analysis

Diets were ground to 1 mm particle size (Wiley Mill, Variable Speed Digital ED-5 Wiley Mill; Thomas Scientific, Swedesboro, NJ) and analyzed in duplicate for DM (method 930.15 [AOAC, 2007]), acid-hydrolyzed ether extract (**aEE**; method 2003.06; AOAC, 2007), and N (method 990.03 [AOAC, 2007]; TruMac; LECO Corp., St. Joseph, MI). An EDTA sample (9.56% N) was used as the standard for calibration and was determined to contain 9.55 ± 0.01% N. Crude protein was calculated as N × 6.25. Gross energy was determined in duplicate using an isoperibolic bomb calorimeter (model 6200; Parr Instrument Co., Moline, IL). Benzoic acid (6,318 kcal GE/kg) was used as the standard for calibration and was determined to contain 6,319 ± 0.8 kcal GE/kg.

Total lipids were extracted by using a chloroform and methanol mixture (Folch et al., 1957). Concentrations of lipid sources were determined gravimetrically. The lipids were methylated directly with acetyl chloride and methanol (Christie, 1972). Fatty acid methyl esters were quantified by a gas chromatograph (Varian 3800, Agilent Technologies, Palo Alto, CA) equipped with a Supelco Sp-2380 column and a flame ionization detector. Gas-chromatograph conditions were as follows: initial column temp, 70 °C with a hold time of 4 min, temperature ramp was 13 °C/min with a final column temperature of 215 °C. Peaks were identified by using commercially available fatty acid methyl ester standards (Nu-Chek-Prep Inc., Elysian, MN).

### RNA isolation and quantitative PCR

Approximately 30 mg of liver tissue was homogenized using the QIAGEN Tissuelyser II (QIAGEN Group; Germantown, MD, USA). Total RNA was isolated using the QIAGEN RNeasy Mini Kit (QIAGEN Group) according to the manufacturer’s recommendations. Approximately 100 mg of adipose tissue was homogenized using the Qiagen Tissuelyser II (QIAGEN Group). Total RNA was isolated using the QIAGEN RNeasy Lipid Tissue Mini Kit (QIAGEN Group) according to the manufacturer’s recommendations. The concentration of RNA was quantified using a spectrophotometer (BioTek Cytation 5; Agilent Technologies, Santa Clara, CA). All samples had 260:280 nm ratios above 1.8. The QuantiTect Reverse Transcription Kit (QIAGEN Group) was used according to the manufacturer’s instructions to synthesize complementary DNA (cDNA) from 0.8 μg of the isolated liver RNA and 0.5 μg of the isolated adipose RNA. All cDNA samples were diluted 10-fold with nuclease-free water.

Real-time quantitative polymerase chain reaction (**PCR**) was performed using iQ SYBR Green Supermix (Bio-Rad Laboratories, Inc., Hercules, CA). The gene-specific primers, shown in Table 4, were diluted to 10 µM with nuclease-free water (Life Technologies; Austin, TX). *Ribosomal protein—L19 (RPL19)* was included as an endogenous reference gene. Each reaction included 10 µL of SYBR Green Supermix, 1 µL of each forward and reverse primer (Integrated DNA Technologies; Coralville, IA), 5 µL of nuclease-free water, and 3 µL of cDNA, for a total of 20 µL reaction volume. Each 96-well plate contained a no-reverse transcriptase negative control and a pooled cDNA reference sample. Samples were assayed in duplicate. Fluorescence of SYBR Green was quantified with a Real-time PCR Detection System (iQ5; Bio-Rad Laboratories Inc.). Cycling conditions were as follows: 5-min initial denaturation at 95 °C followed by 40 PCR cycles (95 °C for 30 s, variable annealing temperature for 30 s, and 72 °C for 30 s; Table 4) and a dissociation curve to verify the amplification of a single PCR product. Optical detection was performed at 55 °C. Analyses of amplification plots were performed with an Optical System Software version 2.0 (iQ5; Bio-Rad Laboratories Inc.) and cycle threshold (**Ct**) values for each reaction were obtained. The mRNA abundance for each sample was normalized to *RPL19* and the pooled sample, and fold change was calculated using the 2^−ΔΔCT^method (Livak and Schmittgen, 2001).

**Table 4.** Primer sequences used for quantitative PCR to assess lipid metabolism

Gene^1^	Primer sequence (5ʹ–3ʹ)^2^	Annealing temperature, °C	Reference
*ACACA*	F: ATGGATGAACCGTCTCCC	51	Kellner et al., 2017
	R: TGTAAGGCCAAGCCATCC		
*CD36*	F: CTGGTGCTGTCATTGGAGCAGT	55	Li et al., 2017
	R: CTGTCTGTAAACTTCCGTGCCTGTT		
*CPT1A*	F: GCATTTGTCCCATCTTTCGT	55	Varady et al., 2012
	R: GCACTGGTCCTTCTGGGATA		
*FABP-1*	F: ACATCAAGGGGACATCGG	55	Kellner et al., 2017
	R: GTCTCCATCTCACACTCC		
*FASN*	F: CACAACTCCAAAGACACG	50	Kellner et al., 2017
	R: AGGAACTCGGACATAGCG		
*HSL*	F: AACGCAATGAAACAGGCC	50	Kellner et al., 2017
	R: TGTATGATCCGCTCAACTCG		
*LPL*	F: CCCTATACAAGAGGGAACCGGAT	55	Li et al., 2017
	R: CCGCCATCCAGTCGATAAACGT		
*PPAR-γ*	F: GTGGAGACCGCCCAGGTTTG	55	Li et al., 2017
	R: GGGAGGACTCTGGGTGGTTCA		
*PPAR-α*	F: AACGGCATCCAGAACAAG	50	Kellner et al., 2017
	R: CATCACAGAGGACAGCATGG		
*RPL 19*	F: AACTCCCGTCAGCAGATCC	55	Li et al., 2019
	R: GTACCCTTCCGCTTACCG		

^1^ *ACACA*, acetyl CoA carboxylase; *CD36,* cluster of differentiation 36/fatty acid translocase; *CPT1A*, carnitine palmitoyl transferase 1 A; *FABP1*, fatty acid binding protein 1; *FASN,* fatty acid synthase; *HSL,* hormone sensitive lipase; *LPL*, lipoprotein lipase; *PPAR-*α, peroxisome proliferator-activated receptor alpha; *PPAR-γ*, peroxisome proliferator-activated receptor gamma; *RPL-19*, ribosomal protein-19.

^2^Direction of primer (F, forward; R, reverse).

### Inflammatory markers

Concentrations of plasma and synovial fluid C-reactive protein (**CRP**), tumor necrosis factor-α (TNFα), and interleukin-6 (IL-6) were quantified using porcine-specific enzyme-linked immunosorbent assay according to the manufacturer’s instructions (R&D Systems, Minneapolis, MN). Plasma and synovial fluid TNFα and IL-6 were analyzed undiluted. Plasma and synovial fluid samples for CRP analysis were diluted 1:20,000 and 1:500, respectively, with provided assay buffer. Plates were read at a wavelength of 450 nm (BioTek Cytation 5; Agilent Technologies), and a coefficient of variation of under 8% between duplicates was deemed acceptable.

### Statistical analysis

Growth performance data by phase were analyzed as repeated measures using mixed model methods of SAS 9.4 (SAS Inst., Cary, NC). Pen was considered the experimental unit, with LA level, LA:ALA, sex, phase, and their interactions included as fixed effects in the statistical model. The first-order autoregression covariance structure was selected for the growth performance repeated measures model ­according to Bayesian information criterion. Initial body weight was fit as a covariate for growth performance data. The spatial power covariance structure was selected for the inflammation parameters repeated measures model according to Bayesian information criterion. Baseline measurements were included as a covariate for all inflammatory markers. Normality and homoscedasticity of the studentized residuals were tested using the UNIVARIATE procedure. Data were reported as least squares means and means separation was done using the PDIFF option. Differences were considered significant if *P* was ≤ 0.05 and a tendency if *P* was >0.05 and ≤0.10.

## Results

Mortality for the overall trial period was 1.0%. Pigs did not display any clinical signs of disease or lameness and remained healthy throughout the entire experimental period. Dietary analysis resulted in similar gross energy and acid-hydrolyzed ether extract between treatments (Tables 1 to 3). Analyzed dietary fatty acid composition was similar to calculated fatty acids from dietary formulation (Table 5). The LA:ALA ratios were slightly higher in the 12:1 and 20:1 treatments and these treatments will be noted as 13:1 and 23:1, respectively, going forward in the text.

**Table 5.** Fatty acid composition of dietary treatments

Analyzed fatty acids	30 g/kg linoleic acid			15 g/kg linoleic acid		
	20:1^1^	12:1	4:1	20:1	12:1	4:1
Phase 1						
C14:0, %	0.02	0.02	0.00	0.10	0.11	0.11
C16:0, %	0.97	0.93	0.74	1.42	1.50	1.41
C16:1, %	0.03	0.03	0.01	0.10	0.10	0.09
C18:0, %	0.26	0.24	0.14	0.90	0.99	0.94
C18:1, %	2.05	2.00	1.78	2.36	2.48	2.37
C18:2, %	3.11	3.12	3.16	1.50	1.50	1.53
C18:3, %	0.14	0.24	0.74	0.06	0.11	0.36
LA:ALA^2^	21.7:1	12.8:1	4.3:1	23.2:1	13.3:1	4.3:1
UFA:SFA^3^	4.2:1	4.5:1	6.3:1	1.7:1	1.7:1	1.8:1
Phase 2						
C14:0, %	0.02	0.02	0.00	0.11	0.12	0.11
C16:0, %	0.98	0.94	0.74	1.46	1.50	1.41
C16:1, %	0.03	0.03	0.01	0.10	0.10	0.09
C18:0, %	0.26	0.24	0.14	0.97	1.02	0.98
C18:1, %	2.06	2.01	1.80	2.40	2.47	2.37
C18:2, %	3.10	3.11	3.16	1.49	1.49	1.52
C18:3, %	0.15	0.24	0.74	0.06	0.11	0.36
LA:ALA	21:1	12.8:1	4.3:1	23.5:1	13.4:1	4.2:1
UFA:SFA	4.2:1	4.5:1	6.3:1	1.7:1	1.6:1	1.8:1
Phase 3						
C14:0, %	0.02	0.01	0.00	0.12	0.12	0.12
C16:0, %	0.98	0.94	0.74	1.47	1.50	1.40
C16:1, %	0.03	0.03	0.01	0.10	0.10	0.08
C18:0, %	0.26	0.24	0.14	1.01	1.04	0.99
C18:1, %	2.06	2.01	1.80	2.43	2.47	2.36
C18:2, %	3.10	3.11	3.15	1.49	1.49	1.51
C18:3, %	0.15	0.25	0.74	0.06	0.11	0.36
LA:ALA	21.1:1	12.6:1	4.3:1	23.7:1	13.5:1	4.2:1
UFA:SFA	4.2:1	4.5:1	6.3:1	1.6:1	1.6:1	1.8:1

^1^Dietary fatty acid ratio as dietary linoleic acid:linolenic acid (LA:ALA): 20:1, 12:1, and 4:1.

^2^LA, linoleic acid; ALA, α-linolenic acid.

^3^SFA, saturated fatty acids; USFA, unsaturated fatty acids.

### Growth performance

There was a tendency for pigs receiving the LLA diets to have a higher BW at days 56 and 84 compared to the HLA diet (*P* *=* 0.088; Table 6). Feed efficiency tended to be improved during days 56 to 84 in pigs fed HLA (*P* = 0.091); however, there were no differences for LA in ADG or ADFI across each phase (*P ≥ *0.229). The LA:ALA did not impact BW (*P *= 0.243). There was a tendency for ADG to be improved during days 28 to 56 (*P =* 0.073); however, ADG was not different in the first or third phases. Dietary LA:ALA did not impact ADFI (*P* = 0.034) or G:F (*P* = 0.203) across phases. For the overall days 0 to 84 experimental period, there was no effect of LA or LA × Ratio for ADG, ADFI, or G:F (Table 7; *P ≥ *0.131). Overall feed intake was increased in pigs fed the 23:1 LA:ALA compared to 13:1, with the 4:1 LA:ALA being intermediate (*P *= 0.010).

**Table 6.** The effects of linoleic acid level and essential fatty acid ratio on growth performance and feed efficiency of grow–finish pigs by weigh period^1^

	Linoleic acid^2^		Fatty acid ratio^3^			SEM	*P*-value^4^^,^^5^				
Item^6^	High	Low	23:1	13:1	4:1		LA	Ratio	Period	LA × period	Ratio × period
BW, kg											
Day 0	41.23	41.01	40.61	41.04	41.64	0.592	0.029	0.032	<0.001	0.088	0.243
Day 28	71.02	71.20	70.87	70.93	71.47						
Day 56	101.31	103.04	102.12	100.70	103.19						
Day 84	127.79	129.12	128.85	127.32	129.15						
ADG, kg											
Days 0 to 28	1.06	1.08	1.07	1.07	1.08	0.016	0.131	0.231	<0.001	0.229	0.073
Days 28 to 56	1.08	1.12	1.11	1.06	1.14						
Days 56 to 84	0.95	0.95	0.95	0.96	0.94						
ADFI, kg											
Days 0 to 28	2.18	2.17	2.20	2.14	2.19	0.035	0.504	0.010	<0.001	0.748	0.333
Days 28 to 56	2.72	2.76	2.73	2.66	2.83						
Days 56 to 84	2.76	2.77	2.76	2.74	2.79						
G:F											
Days 0 to 28	0.49	0.49	0.49	0.50	0.49	0.004	0.376	0.643	<0.001	0.091	0.150
Days 28 to 56	0.40	0.41	0.41	0.40	0.41						
Days 56 to 84	0.35	0.34	0.34	0.35	0.34						

^1^Data are least square means; *n* = 10 pens per treatment with five pigs per pen, totaling 300 pigs; growth calculations included pig days to account for morbidity and mortality.

^2^Calculated dietary linoleic acid: High = 30 g/kg; Low = 15 g/kg.

^3^Calculated fatty acid ratio as dietary linoleic acid:linolenic acid.

^4^Within a dependent variable, means without a common superscript differ significantly (*P < *0.05).

^5^Across all variables, there were no differences for the Period × LA × Ratio interaction.

^6^BW, body weight; ADG, average daily gain; ADFI, average daily feed intake; G:F, gain:feed ratio.

**Table 7.** The effects of linoleic level and essential fatty acid ratio on overall growth performance and feed efficiency of grow–finish pigs^1^

	Linoleic acid^2^		Fatty acid ratio^3^			SEM	*P*-value^4^^,^^5^		
Item^6^	High	Low	23:1	13:1	4:1		LA	Ratio	LA × Ratio
ADG, kg	1.03	1.05	1.05	1.03	1.06	0.011	0.131	0.231	0.845
ADFI, kg	2.54	2.58	2.60^a^	2.52^b^	2.56^ab^	0.036	0.504	0.010	0.504
G:F	0.41	0.41	0.41	0.41	0.41	0.003	0.376	0.643	0.712

^1^Data are least square means; *n* = 10 pens per treatment with five pigs per pen, totaling 300 pigs; growth calculations included pig days to account for morbidity and mortality.

^2^Calculated dietary linoleic acid: High = 30 g/kg; Low = 15 g/kg.

^3^Calculated fatty acid ratio as dietary linoleic acid:linolenic acid.

^4^Within a dependent variable, means without a common superscript differ significantly (*P < *0.05).

^5^For all variables, there were no differences for the interaction of Period × LA × ratio (*P* > 0.10).

^6^ADG, average daily gain; ADFI, average daily feed intake; G:F, gain:feed ratio.

There was a significant effect of sex within each dietary phase and for the overall period (*P < *0.001; Tables 8 and 9), as well as a phase × sex interaction (*P < *0.001). Generally, barrows had increased BW, ADG, ADFI, and reduced feed efficiency compared to gilts. There was a phase × sex × ratio interaction for body weight (*P = *0.001). Barrows fed a 4:1 LA:ALA had increased BW on day 56 compared to 13:1 or 23:1 and increased final BW compared to 23:1, with 13:1 performing intermediately (Table 6; *P *= 0.006). Gilts receiving diets formulated with the 23:1 LA:ALA had increased BW compared to gilts receiving the 4:1 and 13:1 LA:ALA ratios at days 56 and 84. Additionally, gilts fed a 23:1 LA:ALA had improved overall days 0 to 84 ADG compared to gilts receiving the 13:1 diets, with the 4:1 diets performing intermediately (Table 9; *P =* 0.023).

**Table 8.** The effects of linoleic acid level, essential fatty acid ratio, and sex on overall growth performance and feed efficiency of grow–finish pigs^1^

	Linoleic acid^2^				Fatty acid ratio^3^						SEM	*P*-value^4^^,^^5^	
Item^6^	High		Low		23:1		13:1		4:1			Period × Sex × LA	Period × Sex × Ratio
	M	F	M	F	M	F	M	F	M	F			
BW, kg													
Day 0	41.10	41.32	40.73	41.23	40.89^k^	40.33^k^	41.25^k^	40.83^k^	40.61^k^	42.66^k^	1.315	0.660	0.006
Day 28	73.20	68.82	72.58	69.77	72.53^i^	69.20^j^	72.79^i^	69.07^j^	73.34^i^	69.61^j^			
Day 56	105.23	97.37	105.76	99.66	104.36^f^	99.89^g^	104.68^f^	96.71^h^	107.45^e^	98.94^gh^			
Day 84	132.48	123.07	132.63	125.57	130.66^b^	127.04^c^	132.37^ab^	122.27^d^	134.64^a^	123.66^d^			
ADG, kg													
Days 0 to 28	1.15	0.98	1.13	1.04	1.13	1.02	1.13	1.02	1.16	1.00	0.035	0.662	0.512
Days 28 to 56	1.14	1.02	1.18	1.07	1.13	1.08	1.14	0.98	1.21	1.08			
Days 56 to 84	0.98	0.93	0.96	0.93	0.94	0.96	1.00	0.91	0.97	0.92			
ADFI, kg													
Days 0 to 28	2.32	2.03	2.29	2.06	2.31	2.08	2.28	2.00	2.34	2.04	0.073	0.843	0.620
Days 28 to 56	2.95	2.50	2.99	2.54	2.89	2.58	2.90	2.42	3.11	2.55			
Days 56 to 84	2.95	2.57	2.94	2.60	2.88	2.63	2.97	2.52	2.99	2.60			
G:F													
Days 0 to 28	0.49	0.49	0.49	0.50	0.49	0.49	0.50	0.49	0.49	0.50	0.009	0.485	0.609
Days 28 to 56	0.39	0.41	0.40	0.42	0.39	0.42	0.39	0.41	0.39	0.42			
Days 56 to 84	0.33	0.36	0.33	0.35	0.33	0.36	0.34	0.36	0.33	0.35			

^1^Data are least square means; *n* = 10 pens per treatment with five pigs per pen, totaling 300 pigs; growth calculations included pig days to account for morbidity and mortality.

^2^Calculated dietary linoleic acid: High = 30 g/kg; Low = 15 g/kg.

^3^Calculated fatty acid ratio as dietary linoleic acid:linolenic acid.

^4^Within a dependent variable, means without a common superscript differ significantly (*P < *0.05).

^5^For all variables, *P* < 0.01 for period, sex, and the period × sex interaction. There were no differences across variables for the interaction of period × sex × LA × ratio (*P* > 0.10).

^6^M, male; F, female; BW, body weight; ADG, average daily gain; ADFI, average daily feed intake; G:F, gain:feed ratio.

**Table 9.** The effects of linoleic acid level, essential fatty acid ratio, and sex on overall growth performance and feed efficiency of grow–fsinish pigs^1^

	Linoleic acid^2^				Fatty acid ratio^3^						SEM	*P*-value^4^^,^^5^		
Item^6^	High		Low		23:1		13:1		4:1			Sex × LA	Sex × ratio	Sex × LA × ratio
	M	F	M	F	M	F	M	F	M	F				
ADG, kg	1.09	0.98	1.09	1.01	1.07^b^	1.02^c^	1.09^ab^	0.96^d^	1.11^a^	1.00^cd^	0.023	0.200	0.023	0.577
ADFI, kg	2.74	2.36	2.74	2.40	2.69	2.43	2.71	2.31	2.81	2.39	0.065	0.561	0.140	0.573
G:F	0.40	0.42	0.40	0.42	0.40	0.42	0.40	0.42	0.40	0.42	0.006	0.291	0.445	0.776

^1^Data are least square means; *n* = 10 pens per treatment with 5 pigs per pen, totaling 300 pigs; growth calculations included pig days to account for morbidity and mortality.

^2^Calculated dietary linoleic acid: High = 30 g/kg; Low = 15 g/kg.

^3^Calculated fatty acid ratio as dietary linoleic acid:linolenic acid.

^4^Within a dependent variable, means without a common superscript differ significantly (*P < *0.05).

^5^For all variables, there were no differences for the interaction of period × LA × ratio or period × Sex × LA × ratio (*P* > 0.10).

^6^ADG, average daily gain; ADFI, average daily feed intake; G:F, gain:feed ratio.

### Liver and adipose gene transcription

In the liver, there was no effect of dietary LA on gene mRNA abundance (*P ≥ *0.144; Table 10). The 4:1 LA:ALA increased *CD36* compared to the 13:1 LA:ALA, with the high dietary EFA ratio being intermediate (*P *= 0.039). In the adipose tissue, there was no effect of ratio for any genes measured (*P ≥ *0.295; Table 11). Gene expression of *CD36* tended to be greater in pigs fed low LA diets (*P *= 0.086).

**Table 10.** Effect of linoleic acid level and essential fatty acid ratio on liver gene mRNA abundance of finishing pigs

Gene^1^	Linoleic acid^2^		Fatty acid ratio^3^			SEM	*P*-values		
	High	Low	23:1	13:1	4:1		LA	Ratio	LA × ratio
*ACACA*	3.22	3.07	2.80	3.35	3.31	0.203	0.782	0.632	0.285
*CD36*	5.99	5.51	6.07^ab^	4.58^b^	6.82^a^	0.143	0.506	0.039	0.808
*CPT1A*	4.28	2.16	4.11	2.02	3.38	0.554	0.162	0.469	0.016
*FABP1*	3.46	4.11	4.56	3.23	3.65	0.133	0.144	0.056	0.472
*FASN*	2.04	2.23	2.72	1.86	1.92	0.260	0.702	0.320	0.520
*PPARα*	3.60	3.06	3.41	3.28	3.27	0.218	0.397	0.979	0.966

^1^ *ACACA*, acetyl CoA carboxylase; *CD36*, cluster of differentiation 36; *CPT1A*, carnitine palmitoyltransferase 1A; *FABP1*, fatty acid binding protein 1; *FASN*, fatty acid synthase; *PPARα*, peroxisome proliferator-activated receptor alpha.

^2^Calculated dietary linoleic acid: High = 30 g/kg; Low = 15 g/kg.

^3^Calculated fatty acid ratio as linoleic acid:linolenic acid.

**Table 11.** Effect of linoleic acid level and essential fatty acid ratio on adipose gene mRNA abundance of finishing pigs

Gene^1^	Linoleic acid^2^		Fatty acid ratio^3^			SEM	*P*-values		
	High	Low	23:1	13:1	4:1		LA	Ratio	LA × ratio
*ACACA*	1.868	2.461	1.798	2.654	2.091	0.422	0.186	0.317	0.017
*CD36*	3.186	6.564	4.539	4.402	4.203	0.652	0.082	0.987	0.178
*FASN*	1.946	2.031	1.940	2.412	1.743	0.325	0.798	0.295	0.609
*HSL*	3.053	5.363	4.278	3.967	3.602	0.690	0.170	0.939	0.473
*LPL*	4.838	8.543	6.987	7.953	4.677	1.198	0.369	0.756	0.535
*PPARα*	2.908	4.118	2.815	3.274	4.539	0.639	0.296	0.504	0.035
*PPARγ*	2.800	3.702	3.558	2.889	3.195	0.383	0.277	0.797	0.028

^1^ *ACACA*, acetyl CoA carboxylase; *CD36*, cluster of differentiation 36; *FASN*, fatty acid synthase; *HSL*, hormone sensitive lipase; *LPL*: lipoprotein lipase; *PPARα*, peroxisome proliferator-activated receptor alpha; *PPARγ*, peroxisome proliferator-activated receptor gamma.

^2^Calculated dietary linoleic acid: High = 30 g/kg; Low = 15 g/kg.

^3^Calculated fatty acid ratio as linoleic acid:linolenic acid.

### Plasma and synovial fluid inflammatory markers

Linoleic acid, LA:ALA, or their interaction did not impact plasma CRP, TNFα, or IL-6 across each time point (*P ≥ *0.314; Table 12). On day 84, CRP, TNFα, and IL-6 were reduced in the plasma, regardless of diet (*P ≤ *0.037). Similarly, there was no effect of LA, LA:ALA, or their interaction on any markers measured in the hock and carpus synovial fluid (*P ≥ *0.521; Table 13). C-reactive protein and TNFα were reduced in the hock and synovial fluid on day 84, regardless of diet (*P ≤ *0.049).

**Table 12.** Effect of linoleic acid level and essential fatty acid ratio on plasma cytokine concentrations of finishing pigs

Item^4^	Linoleic acid		Fatty acid ratio^1^			SEM	*P*-value^3^					
	High	Low	23:1	13:1	4:1		LA	Ratio	Period	LA ×ratio	LA × period	Ratio × Period
CRP, μg/mL												
Day 0	30.62	33.62	29.77	29.06	37.53	3.200	0.044	0.245	< 0.001	0.430	0.584	0.601
Day 21	23.58	37.07	38.02	21.26	31.69							
Day 42	16.44	24.82	25.17	15.98	20.73							
Day 84	6.64	9.19	8.31	9.17	6.27							
TNFα, pg/mL												
Day 0	30.74	24.13	30.06	22.56	29.79	5.126	0.121	0.881	0.005	0.789	0.675	0.510
Day 21	23.59	17.48	22.00	19.27	21.21							
Day 42	21.11	18.15	16.66	25.38	17.74							
Day 84	15.99	16.52	14.84	17.04	16.97							
IL-6, pg/mL												
Day 0	81.25	78.32	85.21	79.45	75.02	24.002	0.521	0.617	0.037	0.854	0.572	0.315
Day 21	60.98	69.05	67.73	59.02	70.41							
Day 42	50.27	48.22	51.05	49.93	51.42							
Day 84	28.98	31.23	29.78	33.29	40.87							

^1^Calculated dietary linoleic acid: High = 30 g/kg; Low = 15 g/kg.

^2^Calculated fatty acid ratio as linoleic acid:linolenic acid.

^3^For all variables, there was no effect for the interaction of period × LA × ratio (*P *≥ 0.10).

^4^CRP, C-reactive protein; TNFα, tumor necrosis factor-alpha; IL-6, interleukin-6.

**Table 13.** Effect of linoleic acid level and essential fatty acid ratio on synovial fluid cytokine concentrations of finishing pigs

	Linoleic acid^1^		Fatty acid ratio^2^			SEM	*P*-value^3^			
Item^4^	30 g/kg	15 g/kg	23:1	13:1	4:1		Period	LA × Ratio	LA × Period	Ratio × Period
Hock										
CRP, ng/mL										
Day 0	1535.09	1649.42	1668.29	1486.42	1622.06	186.92	< 0.001	0.395	0.976	0.971
Day 84	727.73	832.60	894.94	623.42	822.13					
TNFα, pg/mL										
Day 0	32.44	36.55	31.78	33.02	32.19	5.845	0.046	0.845	0.641	0.784
Day 84	20.87	22.42	21.45	22.24	21.89					
IL-6, pg/mL										
Day 0	28.57	26.89	28.01	29.56	26.12	6.589	0.115	0.888	0.702	0.213
Day 84	24.12	25.01	21.12	22.65	21.87					
Carpus										
CRP, ng/mL										
Day 0	1695.45	1516.33	1674.66	1581.02	1602.63	191.88	< 0.001	0.412	0.844	0.739
Day 84	788.55	624.21	837.50	718.14	798.82					
TNFα, pg/mL										
Day 0	32.55	30.48	31.98	36.87	34.12	6.994	0.049	0.957	0.659	0.711
Day 84	20.92	19.68	18.71	22.22	22.45					
IL-6, pg/mL										
Day 0	31.48	33.45	35.12	29.61	30.29	6.512	0.257	0.867	0.911	0.636
Day 84	25.74	23.41	23.48	20.08	23.99					

^1^Calculated dietary linoleic acid: High = 30 g/kg; Low = 15 g/kg.

^2^Calculated fatty acid ratio as linoleic acid:linolenic acid.

^3^For all variables, there was no effect for the interaction of LA, ratio, or period × LA × ratio (*P *≥ 0.10).

^4^CRP, C-reactive protein; TNFα, tumor necrosis factor-alpha; IL-6, interleukin-6.

## Discussion

Linoleic acid and ALA have been shown to be important in lactating sow diets (Rosero et al., 2016); though limited research exists in growing pigs evaluating dietary LA:ALA. This study evaluated the effects of dietary LA:ALA and ­linoleic acid inclusion based on different combinations of corn oil, flaxseed oil, choice white grease, and beef tallow on the growth performance, lipid metabolism, and inflammation in grow–finish pigs. Dietary linoleic acid inclusion of 30 g/kg was utilized to achieve similar LA intake to the 125 g/d LA intake recommended by Rosero et al., (2016) in lactating sows. Inclusion of linoleic acid at 15 g/kg was selected based on the same author’s recommendation of 10 g/d of ALA, which was achieved at the 4:1 LA:ALA. Linoleic acid intake of pigs fed the HLA diets was approximately 68, 84, and 86 g/d during phases 1, 2, and 3, respectively. Intake of LA in pigs receiving LLA diets was approximately 33, 41, and 42 g/d for phases 1, 2, and 3, respectively. Pigs receiving the low LA diet formulated to a 4:1 LA:ALA had an ALA intake of approximately 9 g/d across the overall period.

It is a commonly accepted that barrows have increased gain and reach market weight faster compared to gilts, and that gilts are more feed efficient compared to barrows (Friesen et al., 1994; Smit et al., 2014). Results of this study would agree with these findings, as the effect of sex was significant across all growth performance parameters. However, gilts in this study had improved final BW when fed the 13:1 LA:ALA diets, though this increased final BW was still less than barrow final BW. This increase in final BW of gilts was a result of increased overall ADG. Similar results of increased BW were reported by Becker et al. (2023) in gilts fed ­varying dietary LA:ALA ratios; however, this was observed when pigs were fed a 13:1 LA:ALA, rather than a 23:1 LA:ALA. Diets between the two studies utilized the same fat sources; however, dietary fat inclusion and therefore, total fatty acid content differed. The authors hypothesized that this change in gilt growth may be due to delaying the onset of puberty in these females; however, further work describing the underlying mechanisms is needed. In pigs fed 2% added canola oil, similar ADG between growing gilts and barrows was reported; however, this observation did not occur in pigs fed 5% canola oil or 2% tallow (Dugan et al., 2004). Canola oil has an approximate LA:ALA of 5:1 (NRC, 2012), which contrasts the data presented herein, as there was no growth performance benefit in feed a 4:1 LA:ALA. Additional, large-scale commercial studies are needed to further elucidate this effect of LA:ALA on growth of barrows and gilts.

In the pig, de novo lipogenesis occurs in the adipose tissue (O’Hea and Leveille, 1969), which contrasts humans and rodents where it occurs in the liver. In the current study, *CD36* expression was increased in the liver of pigs fed the 4:1 LA:ALA, regardless of linoleic acid inclusion. The *CD36* gene encodes for cluster of differentiation 36, also known as fatty acid translocase, and is responsible for uptake of long-chain FA. Overall intake of long-chain FA was slightly increased in pigs receiving the 4:1 diets compared to the other two LA:ALA ratios. Previously published literature in rodents also demonstrated an increase in *CD36* abundance in the plasma membrane of skeletal muscle (Chorner et al., 2016) and adipose tissue due to dietary ALA supplementation (Wang et al., 2016). Conversely, expression of *CD36* was also increased in the adipose tissue of pigs receiving diets with low LA. Oleic acid was high in the low LA, which has been shown to up-­regulate *CD36* expression in ovarian granulosa cells (Zhou et al., 2022), but this effect remains to be elicited in other tissue types in pigs. Several interactions were observed in the gene expression data in the current study. These interactions of dietary LA inclusion and LA:ALA on lipid metabolism in growing pigs require further investigation, as the data presented herein are inconclusive.

Metabolism of LA and ALA leads to the production of various pro- and anti-inflammatory compounds (Fritsche, 2015). Dietary LA inclusion or LA:ALA did not influence markers of inflammation either systemically or locally in the joint. As a result, inflammatory markers remained relatively low across all collection points. C-reactive protein, IL-6, and TNFα were reduced on day 84 compared to day 0 in the plasma. This reduction in inflammation could have been due to decreases in protein gain and growth rate typically experienced in late-finishing pigs (van der Most et al., 2011). C-reactive protein is an acute phase protein used in veterinary diagnostics to evaluate health status of the swine herd. Normal ranges for serum CRP are 5 to 30 µg/mL, which is consistent with reported observations in the current study (Pomorska-Mól et al., 2012). Interleukin-6 has been shown to play a role in cartilage degradation in murine models (Jazayeri et al., 2010). Tumor necrosis factor-alpha is a pro-inflammatory cytokine responsible for activating the adaptive immune system. Circulating levels of IL-6 and TNFα observed are similar to those previously reported in healthy pigs, though minimal data exists regarding normal levels of these markers in synovial fluid of healthy pigs.

In conclusion, gilts fed a 23:1 dietary LA:ALA had increased final BW compared to gilts fed a 13:1 or 4:1 LA:ALA ratio, which was a result of increased overall gain. Regardless of diet, some pro-inflammatory markers were reduced with time, both systemically and locally in the joint. Overall, differing LA:ALA ratios can be fed at either 15 g/kg or 30 g/kg LA without influencing growth, as there were no LA × LA:ALA interactions observed. The data presented does not support lowering the LA:ALA to 4:1, as previously reported in the literature (Calder, 2008; Duan et al., 2014; Szostak et al., 2016), for reducing inflammation in growing pigs. However, a 4:1 dietary LA:ALA appears to be beneficial for improving growth in barrows. Furthermore, LA:ALA ratios can differentially impact the growth of gilts, which may be a result of delaying puberty onset. To understand the biological mechanisms behind the improved gilt growth performance observed, research investigating the hormone production and reproductive characteristics in growing gilts fed varying LA:ALA is warranted.

## Abbreviations:

ACACA: acetyl CoA carboxylase
ADG: average daily gain
ADFI: average daily feed intake
aEE: acid ether extract
ALA: α-linolenic acid
ARA: arachidonic acid
BW: body weight
CD36: cluster of differentiation 36/fatty acid translocase
CLA: conjugated LA
CPT1A: carnitine palmitoyl transferase 1 A
CRP: C-reactive protein
Ct: cycle threshold
DGLA: dihomo-γ-linolenic acid
DHA: docosahexaenoic acid
EDTA: ethylenediaminetetraacetic acid
EFA: essential fatty acids
EPA: eicosapentaenoic acid
FA: fatty acid
FABP1: fatty acid binding protein 1
FASN: fatty acid synthase
G:F: gain-to-feed ratio
GE: gross energy
GLA: γ-linolenic acid
HSL: hormone sensitive lipase
IL-6: interleukin-6
LA: linoleic acid
LPL: lipoprotein lipase
ME: metabolizable energy
MUFA: monounsaturated fatty acids
PCR: polymerase chain reaction
PPAR-α: peroxisome proliferator-activated receptor alpha
PPAR-*γ*: peroxisome proliferator-activated receptor gamma
PUFA: polyunsaturated fatty acid
RPL-19: ribosomal protein-19
SFA: saturated fatty acids
SID: standardized ileal digestible
STTD: standardized total tract digestible
TNFα: tumor necrosis factor-α
